# Perioperative analgesic effects of an ultrasound-guided transversus abdominis plane block using bupivacaine in goats undergoing celiotomy

**DOI:** 10.3389/fvets.2023.1197728

**Published:** 2023-11-24

**Authors:** Tate B. Morris, Klaus Hopster, Marie-Eve Fecteau

**Affiliations:** Department of Clinical Studies, New Bolton Center, University of Pennsylvania, Kennett Square, PA, United States

**Keywords:** locoregional, anesthesia, nociceptive, transversus abdominis plane (TAP), celiotomy, goat

## Abstract

**Introduction:**

Never has the anatomy, the procedure of the transversus abdominis plane (TAP) block, or the perioperative analgesic effects of a bupivacaine TAP block been described in goats.

**Methods:**

This report details the relevant anatomy in a cadaveric study combined with the description/use of a TAP block in a controlled, randomized, prospective, blinded clinical study in which 20 goats with urolithiasis presenting for either ventral midline or paramedian celiotomy were enrolled. Anesthesia was induced with ketamine and midazolam and maintained with desflurane in oxygen. An ultrasound-guided TAP block was performed using 0.25% bupivacaine (4 sites, 0.4 mL/kg each site) (bupivacaine-TAP, *n* = 10) or equal volume of saline (control-TAP, *n* = 10). When indicated, urethral amputation was performed followed by celiotomy with cystotomy or tube cystostomy. Urethrotomy was performed if warranted. Intraoperatively, a 20% increase in mean arterial pressure (MAP), heart rate (HR) and/or respiratory frequency was treated with an increase in desflurane concentration of 0.5 Vol.%. Goats received ketamine boluses (0.2 mg/kg IV) when moving spontaneously. At 2, 12, and 24 h post-extubation, pain was scored with a descriptive scale. Data were analyzed with an analysis of variance (ANOVA) or the Wilcoxon signed-rank test, and *P* < 0.05 was considered statistically significant.

**Results:**

Bupivacaine-TAP goats exhibited lower end-tidal desflurane concentration requirements (*P* = 0.03), lower pain scores at 2-h post-extubation (*P* = 0.02), shorter anesthetic recovery times (*P* = 0.03) and decreased HR and MAP during surgical stimulation. Goats receiving a bupivacaine TAP block experienced less intraoperative nociceptive input requiring less inhalant anesthetic leading to faster anesthetic recoveries and decreased postoperative pain.

**Discussion:**

Ultrasound-guided TAP block is a simple technique to decrease anesthetic requirement while providing additional postoperative comfort in goats undergoing celiotomy.

## 1 Introduction

Small ruminants, especially goats, are increasing in popularity as pets and show animals, and therefore being evaluated and treated in large animal hospitals more frequently. Urolithiasis is a multifactorial disease with anatomical, dietary and husbandry predispositions that make it the most common urinary tract disease in small ruminants (1). Uroliths typically form in the urinary bladder and when voided may cause life-threatening partial or complete blockage of the urethra resulting in secondary urinary tract rupture, ureteral or renal damage (1). Additionally, the post-renal obstruction causes systemic buildup of normally excreted nitrogenous waste and/or electrolytes, particularly potassium, which in turn can result in fatal arrhythmias or uremic encephalopathy. Given a high initial failure or recurrence of obstruction with either medical management using glacial acetic acid (Walpole's solution) or simple surgical urethral process amputation (2, 3) urolith removal via cystotomy combined with urohydropulsion is often required for long-term success (3). This procedure is frequently coupled with placement of an indwelling Foley catheter within the bladder (tube cystostomy) to temporarily bypass the urethra postoperatively (1).

Analgesia in these patients can be challenging. There is no medication specifically approved for analgesic use in small ruminants in the United States; therefore, whether a pet or food-producing animal, all use is extra-label and residue implications must be considered (4). A mainstay of pain management in small ruminants has been the use of non-steroidal anti-inflammatory drugs (NSAIDs) and opioid medications (5). NSAIDs are crucial to both pain control and reduction of inflammation through decreased prostaglandin synthesis; however, possible side effects such as gastrointestinal ulceration, and renal toxicity can make them contraindicated in the face of azotemia and potential renal impairment encountered with urinary obstruction (6). Opioids like morphine, fentanyl, butorphanol or buprenorphine are used to control intra- and postoperative pain in small ruminants, but can decrease gastrointestinal motility, cause ataxia and result in CNS excitation (5). Furthermore, although provisioned in the Animal Medicinal Drug Use Clarification Act (AMDUCA) of 1994, small ruminants are classified in the United States as minor species by the Federal Drug Administration and therefore extra-label use of opioids requires consideration in individual case management. Given these implications, alternative means for analgesia provision should be investigated.

Locoregional anesthetic techniques have gained widespread practice in veterinary anesthesia for the management of perioperative pain (7). The main goals of locoregional anesthesia are to provide preventive and multimodal perioperative analgesia in combination with other analgesic drugs to decrease the stress response to surgical trauma, to reduce the potential development of central sensitization and to reduce the anesthetic requirements and autonomic responses to surgical stimuli (8, 9). Such effects are achieved through transmission inhibition of myelinated Aδ and unmyelinated C fibers nociceptive input via voltage-gated Na^+^ channel blockade. Bupivacaine hydrochloride is a commonly used Na^+^ channel blocker in veterinary medicine that exhibits a longer duration of action than lidocaine and mepivacaine (5).

The use of Na^+^ channel blocker epidural anesthesia techniques for goats undergoing abdominal surgery has been described in the literature since the 1980′s with varying success. Such techniques are relatively easy to perform and require neither advanced imaging techniques or specialized equipment; however, there have been several associated complications described including insufficient nociceptive blockade, prolonged recumbency due to motor blockade, ruminal tympany and hypothermia (10–13). A more recently described promising loco-regional anesthesia technique is the interfascial transversus abdominis plane (TAP) block in which local anesthetic is deposited into the fascial plane between the obliquus internus abdominis and transversus abdominis muscles thereby anesthetizing the ventral branches of the thoracic and lumbar spinal nerves (14, 15). It results in nociceptive blockade of the abdominal wall including the cutis, subcutaneous fascias, muscles, and parietal peritoneum (16, 17); however, like the other described techniques the blockade does not extend to the abdominal viscera. The TAP block is performed bilaterally for efficacy on ventral midline. In humans, it is indicated for a wide range of abdominal procedures such as cesarean section (18), hysterectomy (19), appendectomy (20), and cholescystectomy (21). In veterinary anesthesia, the TAP block has been described in dogs (16, 22), cats (23), rabbits (24), zoo species (25), calves (26), and ponies (27, 28) for a combination of procedures requiring surgical approaches to the abdomen or mastectomy.

No previous study has investigated the abdominal wall anatomy of the goat with the aim of designing a TAP block for this species; furthermore, no study has evaluated the influence of the TAP block in terms of perioperative analgesia (as systemic analgesic requirement) and inhalant anesthetic sparing effect. More specifically, the aim of this prospective analysis was to describe and evaluate the procedure in goats undergoing midline or paramedian celiotomy for obstructive urolithiasis. We hypothesized that goats receiving a TAP block with bupivacaine would have lower intraoperative anesthetic requirements and lower postoperative pain scores compared to those not receiving the block.

## 2 Materials and methods

### 2.1 Anatomical study

The use of a cadaver for this part of the study did not require ethical review or approval as per the Pennsylvania Institutional Animal Care and Use Committee. The fresh cadaver of a 17-kilogram goat euthanized for reasons unrelated to the study with no history of prior trauma or celiotomy to the area of anatomic interest was dissected. The specimen was positioned in left lateral recumbency, a skin incision was made just caudal to the shoulder on dorsal midline in a straight line extending to ventral midline. A similarly oriented incision was made at the level of the tuber coxae. The two incisions were then connected by a third incision parallel and along dorsal midline. The cutis, subcutaneous fascias and cutaneous trunci muscle were reflected ventrally to midline allowing exposure of the right flank, hemithorax and paramedian areas. The layers of anatomical structures were sequentially dissected and photographed to characterize the innervation of the body wall at the level of a ventral midline or paramedian celiotomy incision. The approach was vital in identifying the ventral spinal nerve branches lying within the transversus abdominis fascial plane for local anesthetic infiltration.

### 2.2 Evaluation of TAP block clinical efficacy

#### 2.2.1 Study design

Controlled, randomized, blinded clinical study.

#### 2.2.2 Animals

Twenty, client-owned goats of various breeds and gender were included in the study. All goats were presented to the Widener Hospital of the School of Veterinary Medicine, University of Pennsylvania for surgical treatment of obstructive urolithiasis. The present study was approved by the Pennsylvania Institutional Animal Care and Use Committee (No. 806893-aaefebh).

#### 2.2.3 Preopertative treatment

After a diagnosis of obstructive urolithiasis was confirmed and informed owner consent obtained, an intravenous jugular catheter was placed. Preoperative antimicrobials consisting of penicillin G procaine (22,000 IU/kg subcutaneously q12 hours, PenOne Pro™, VetOne^Ⓡ^), ceftiofur sodium (2.2 mg/kg subcutaneously q24 hours, Naxcel^Ⓡ^, Zoetis), or a combination were administered at the senior clinician's discretion based on spectrum of bacterial coverage with consideration of extra-label drug use as outlined by AMDUCA in minor food-producing species. A plasma biochemical profile was obtained for each goat during the initial evaluation. If the reported blood creatinine was within the recognized normal caprine reference range of 1.0–1.8 mg/dL (29), preoperative flunixin meglumine (1.1 mg/kg intravenously, Flunixin Injection, Norbrook Laboratories Limited) was administered.

#### 2.2.4 Anesthetic protocol

All goats were classified using a modification of the current American Society of Anesthesiologists (ASA) physical status classification scheme adopted for veterinarian medicine (I-V, including an E to denote emergent case no matter class) (30). After sedation with midazolam (0.5 mg/kg intravenously, Midazolam Hydrochloride 50 mg/10 mL, West-Ward, Inc.), general anesthesia was induced with ketamine (3 mg/kg intravenously, Ketamine Hydrochloride, Covetrus North America) and goats were orotracheally intubated in sternal recumbency and the endotracheal tube connected to a small animal anesthesia machine with a circle rebreathing circuit. Anesthesia was maintained using desflurane (Suprane, novaplus^Ⓡ^) in 100% oxygen and expiratory desflurane concentration (E_T_Des) was targeted to be 6.5 Vol.% and adjusted to maintain an appropriate plane of anesthesia as described below.

Thereafter, the goats were placed in right lateral recumbency on a heated mat for placement of an arterial catheter into the auricular artery. Temperature was measured using an intra-esophageal probe. No goat showed signs of hypothermia and temperature reading was >96°F (35.6°F) in all animals. All animals were instrumented with a three-lead electrocardiograph, pulse oximeter and capnograph. Heart rate (HR) and rhythm, direct arterial pressure, respiratory frequency (R*f* ), hemoglobin saturation of oxygen and end-tidal CO_2_ (E_T_CO_2_) were routinely monitored at 5 min intervals. Goats were initially allowed to breathe spontaneously but were ventilated if indicated by the E_T_CO_2_ (>65 mmHg) using intermittent positive pressure ventilation using a tidal volume of 10 mL/kg and an inspiratory to expiratory ratio of 1:3 with the respiratory rate adjusted to effect to maintain E_T_CO_2_ between 35 and 45 mmHg. Dobutamine (DOBUTamine Hydrochloride, Hospira, Inc) infusion was administered and titrated to effect to maintain mean arterial pressure (MAP) > 65 mmHg. All goats received isotonic crystalloid fluids intravenously at a rate of 8–10 mL/kg/h.

Once a sufficient and safe plane of anesthesia was reached (modified Guedel's anesthetic depth stage III, plane 2–3) (31), goats were placed in dorsal recumbency for preparation of the surgical field. A 20% increase in blood pressure from presurgical baseline, heart rate and/or respiratory frequency during surgical manipulation was treated with an increase in desflurane concentration of 0.5 Vol.%. Goats received a bolus of ketamine (0.2 mg/kg intravenously) when moving spontaneously. General anesthesia and anesthetic monitoring was performed by licensed veterinary technicians trained in general anesthesia, anesthesia residents in training and/or board-certified veterinary anesthesiologists all of whom were blinded to the intervention assignment.

#### 2.2.5 Transversus abdominis plane block

An ultrasound-guided TAP block was performed in all goats by a single injector (author TM) blinded to intervention assignment by injecting either 0.5% bupivacaine HCl (4 mg/kg interfascial total body dose, 0.5% Bupivacaine Hydrochloride Injection 150 mg/30 mL, novaplus^Ⓡ^) diluted to an injectable suspension of final concentration of 0.25% using sterile 0.9% saline (0.9% Sodium Chloride Injection, Fresenius, Kabi) (bupivacaine-TAP, *n* = 10) or equivalent volume of sterile 0.9% saline (control-TAP, *n* = 10). Equivalent injectate volumes of 0.4 mL/kg per application site (two cranial, two caudal) were used (26). An electronic randomizer (Research Randomizer, https://www.randomizer.org/) was used to generate the allocation sequence of intervention group assignments. This sequence was then concealed from the injector (author TM) by use of preprepared numbered envelopes containing nondescript vials of injectate of sufficient volume to perform a TAP block as outlined.

Following aseptic preparation, a 4 to 12 MHz linear ultrasound probe (Phillips Lumify L12-4 broadband linear array transducer) connected to a tablet computer (Samsung Galaxy Tab S4, model SM-T830) for image display was positioned immediately caudal to the last rib, mid-way between the transverse processes of the lumbar vertebrae and ventral midline, and parallel to the long axis of the body. Simultaneous observation of the three layers of the abdominal wall at this level including the obliquus externus abdominis muscle, obliquus internus abdominis muscle, and transversus abdominis muscle was made (or two layers including the rectus abdominis muscle and transversus abdominis muscle). A 20-gauge 2.5″ spinal needle (BD Spinal Needle, Becton, Dickinson, and Company) was passed just through the skin in a craniocaudal direction and attached to the appropriately sized syringe for the injectate volume. As a single operator holding both the probe and needle/syringe, the needle was advanced in a craniocaudal direction beneath the long axis of the ultrasound probe using an in-plane technique (26). When the interfascial plane located between the obliquus internus abdominis muscle (or rectus abdominis muscle) and transversus abdominis muscles was reached, a test dose of 1 mL was injected to confirm placement. If inappropriate hydrodissection (i.e., intramuscular or wrong plane) was observed, the needle was adjusted to ensure positioning in the correct plane. When hydrodissection was observed in the appropriate transversus abdominis fascial plane, a total of 0.4 mL/kg of solution was injected before the needle was withdrawn. Moving caudally, the ultrasound probe was repositioned just cranial to the passively flexed ipsilateral stifle (and/or tuber coxae), midway between the transverse processes of the lumbar vertebrae and ventral midline, and parallel to the long axis of the body. Using a similar technique, an additional 0.4 mL/kg of solution was deposited within the transversus abdominis fascial plane. The procedure was then repeated in an identical manner on the contralateral hemiabdomen.

#### 2.2.6 Surgical procedure(s)

Following TAP block the urethral process was amputated in standard fashion if not previously performed, followed by retrograde passage of an appropriately sized polypropylene urinary catheter to the level of obstruction or urethral recess by a surgeon blinded to the treatment group. Subsequently, a routine caudal paramedian or ventral midline celiotomy incision was made depending on the gender of goat and a cystotomy and/or tube cystostomy completed as previously described (32). If warranted and at the surgeon's discretion, a urethrotomy was performed (32). Surgical procedures were performed by surgical residents in training and/or board-certified veterinary specialists.

#### 2.2.7 Recovery

All goats were recovered with the same recovery environment. Upon completion of surgery, they were placed in sternal recumbency and extubated upon spontaneous swallowing in avoidance of chewing which can result in damage to the orotracheal tube and inadvertent aspiration. At this point flumazenil (0.02 mg/kg intravenously, Flumazenil Injection, novaplus^Ⓡ^) was administered by the anesthetist. Recovery time (in minutes from the end of volatile anesthesia/turning off the vaporizer until the goat was standing) and recovery quality (using a simple numeric scale from 1 to 10) were recorded.

#### 2.2.8 Initial postoperative management

Beginning immediately postoperatively, analgesia was maintained using a combination of flunixin meglumine (1.1 mg/kg intravenously q12 hours), morphine (0.1 mg/kg intramuscularly q8 hours, Morphine Sulfate Injection, hikma^Ⓡ^), phenazopyridine (4 mg/kg orally q12 hours, Phenazopyridine Hydrochloride 95mg, AmerisourceBergen^Ⓡ^), dexamethasone (0.1 mg/kg intravenously once, Dexamethasone Injection 2 mg/mL, PHEONIX™) (33) when appropriate and based on surgeon preference. Goats were maintained on antimicrobials as described preoperatively for the duration of indwelling Foley catheter placement. A gastroprotectant (pantoprazole, 1 mg/kg intravenously or subcutaneously q24 hours, Pantoprazole Sodium for Injection, Northstar Rx LLC) and/or neuroprotectant (thiamine, 10 mg/kg subcutaneously q24 hours, Thiamine Hydrochloride, NEOGEN^Ⓡ^ Vet) were administered based on surgeon preference. In the event of preoperative azotemia, intravenous fluid therapy with necessary supplementation was maintained until biochemical profile revealed normalization of renal values.

#### 2.2.9 Pain scoring

**Figure 1:**
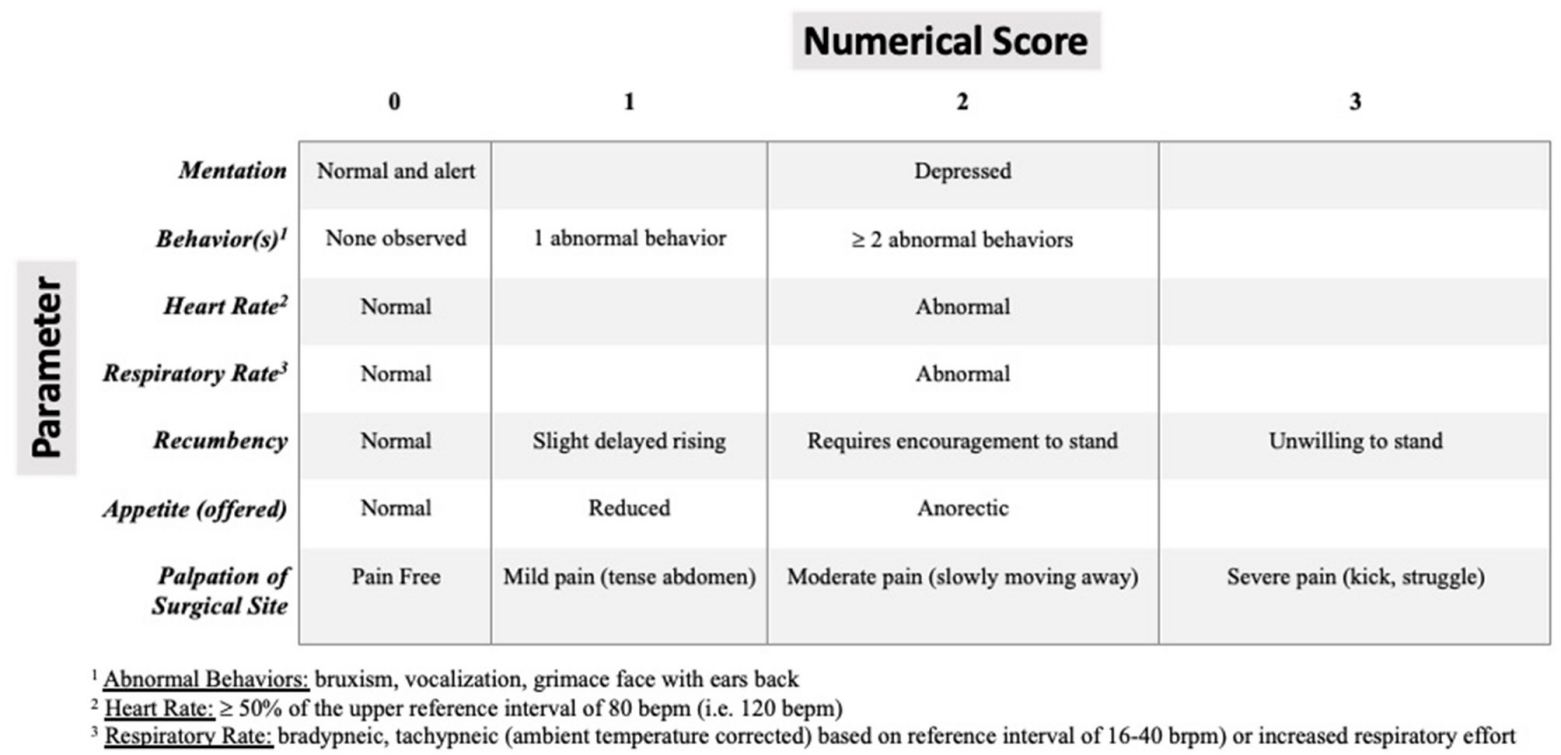
Modified pain assessment scoring system in small ruminants extrapolated from current peer-reviewed literature in goats for reference (33, 35, 36). Zero is normal and 16 is the highest cumulative abnormal score.

Using current peer-reviewed literature in goats for reference (34–36), a simple modified descriptive scale assessing a combination of the following criteria: mentation, painful behavior, heart rate, respiratory rate, recumbency, appetite and palpation of surgical site was created (Figure 1). At 2, 12, and 24 h post-extubation, pain was scored in all goats by a single observer (author TM) blinded to the intervention groups. Each individual criterion was scored. Then a cumulative criteria score out of a maximum of 16 was calculated and recorded at each time point.

#### 2.2.10 Statistical analysis

Analysis was performed using the statistical software program SAS 9.3 (SAS Institute Inc., NC, USA) and GraphPad Prism Version 7 (GraphPad Software, Inc. USA).

A sample size calculation was performed a priori (power of 0.8 and an α-error of 0.05) showing that 10 goats per group would be necessary to detect clinically significant differences in volatile anesthetic requirements and pain scores.

Visual assessment of qq-plots and the Shapiro-Wilk test was used to confirm normal distribution of model residuals of dependent variables.

For heart rate, intraoperative blood pressure and end-tidal percent desflurane requirement the mean over the whole anesthetic period for each animal was calculated (sum of measurements/number of measurements) to eliminate differences in length of anesthetic duration and compared using Student's *t*-test.

Further, length of anesthetic procedures varied but only time periods with a number of goats greater than three were analyzed. A two-way analysis of variance and Tukey's *post hoc* test were used for comparing the measured parameters between the groups by period of time (repeated measurements).

Non-parametric data was analyzed by the Kruskal-Wallis test, followed by the Wilcoxon-Mann-Whitney test, if significance level was reached.

Significance level was set at alpha = 5% (*P* < 0.05). Normally distributed data is presented as mean ± SD and non-parametric data as median (range).

## 3 Results

### 3.1 Anatomical study

Following reflection of the cutis, subcutaneous fascias and cutaneous trunci musculature, the obliquus externus abdominis and rectus abdominis muscles were visible. The obliquus externus abdominis muscle was reflected and removed to expose the obliquus internus abdominis muscle overlying the transversus abdominis muscle. The ventral branches of the thoracic and lumbar spinal nerves were visible running in a dorsal to ventral orientation deep to the obliquus internus abdominis muscle yet superficial to the transversus abdominis muscle within the transversus abdominis fascial plane prior to coursing deep to the rectus abdominis muscle. The rectus abdominis muscle was reflected ventrally and the paths of the ventral branches of the spinal nerves within the transversus abdominis plane were traced to their termination within the deep surface of the rectus abdominis muscle.

### 3.2 TAP block comparison

A total of 20 goats (10 bupivacaine-TAP, 10 control-TAP) that presented for surgical treatment of obstructive urolithiasis at the Widener Hospital of the School of Veterinary Medicine, University of Pennsylvania between October 2019 and August 2020 were enrolled in the study.

Descriptive statistics of the demographic data of the 10 bupivacaine-TAP and 10 control-TAP goats are referenced in Tables 1, 2, respectively. Of note, a urethrotomy was performed in a single goat from each group and a ventral midline celiotomy was performed in the sole female goat. Two goats had undergone a previous caudal paramedian celiotomy during a prior hospitalization (ipsilateral control-TAP, contralateral bupivacaine-TAP). No significant difference in preoperative creatinine values between the bupivacaine-TAP (5.8 ± 4.8 mg/dL) or control-TAP (4.3 ± 3.9 mg/dL) was noted (*P* = 0.47).

**Table 1:** Descriptive demographic data of treatment animals receiving a tranversus abdominis plane (TAP) local anesthetic block with bupivacaine while undergoing celiotomy for obstructive urolithiasis.

**Group TAP**	**Age (yr)**	**Breed**	**Sex**	**Weight (kg)**	**Procedure**	**2 h PE score**	**Analgesia**	**12 h PE score**	**Analgesia**	**24 h PE score**	**Analgesia**
Animal 1	2	Boer	Wether	63	UP amputation, tube cystostomy	2	M	4	M	2	M
Animal 2	2.5	Nigerian Dwarf	Wether	31	UP amputation, tube cystostomy	2	FM, Dx	3	FM	2	FM, Dx
Animal 3	3	Pygmy	Wether	34	UP amputation, tube cystostomy	4	M, P	4	M, P	3	M, P
Animal 4	1	Boer	Wether	89	UP amputation, tube cystostomy	0	M, P	3	M, P	2	M, P
Animal 5	0.42	Boer	Buck	24	Cystotomy	2	M, P	1	M, P	1	M, P
Animal 6	7	Nigerian Dwarf	Doe	44	Cystotomy	4	FM, P	3	FM, P	4	FM, P
Animal 7	0.25	Pygmy	Buck	10	UP amputation, tube cystostomy	3	FM, P	6	FM	5	FM
Animal 8	0.42	Pygmy	Wether	12	UP amputation, tube cystostomy	9	M, P	10	M, P	2	FM, P
Animal 9	8	Nigerian Dwarf	Wether	36	Repeat tube cystostomy, urethrotomy	4	M, P	3	M, P	3	M, P
Animal 10	0.33	Nigerian Dwarf	Wether	13	UP amputation, tube cystostomy	2	M, P	6	M, P	3	M, P
Mean	2.5			35.6							
Median						3		3		3	
SD	2.8			24.8							
Min	0.25			10		0		1		1	
Max	8			89		9		10		5	

Dx, dexamethasone; FM, flunixin meglumine; M, morphine; P, phenazopyridine; PE, post-extubation; UP, urethral process.

**Table 2:** Descriptive demographic data of control animals receiving a tranversus abdominis plane (TAP) block with saline while undergoing celiotomy for obstructive urolithiasis.

**Group CTR**	**Age (yr)**	**Breed**	**Sex**	**Weight (kg)**	**Procedure**	**2 h PE score**	**Analgesia**	**12 h PE score**	**Analgesia**	**24 h PE score**	**Analgesia**
Animal 1	4	Nubian	Wether	81	Tube cystostomy, urethrotomy	9	FM	7	FM, M, P	5	FM, P
Animal 2	3	Pygmy	Wether	31	Cystostomy	8	FM	6	FM	2	FM
Animal 3	0.67	Pygmy	Wether	17	Tube cystostomy	4	M, P	5	M, P	3	FM, P
Animal 4	2	Boer	Wether	88	UP amputation, tube cystostomy	4	FM, P	3	FM, P	3	FM, P
Animal 5	4	Nigerian Dwarf	Wether	32	UP amputation, tube cystostomy	7	M, P	3	M, P	3	FM, P
Animal 6	6	Nigerian Dwarf	Wether	38	UP amputation, tube cystostomy	4	M, P	3	M, P	3	M, P
Animal 7	0.33	Nigerian Dwarf	Wether	11	Tube cystostomy	10	M	6	M	6	M
Animal 8	5	Alpine	Wether	82	Repeat tube cystostomy	9	M, P	2	M, P	1	M, P
Animal 9	11	La Mancha	Wether	78	Tube cystostomy	8	FM, P	9	FM, P	5	FM, M, P
Animal 10	1	Nubian	Wether	28	UP amputation, tube cystostomy	7	FM	5	FM	4	FM
Mean	3.7			48.6							
Median						7		5		3	
SD	3.2			30.1							
Min						4		2		1	
Max						10		9		6	

FM, flunixin meglumine; M, morphine; P, phenazopyridine; PE, post-extubation; UP, urethral process.

**Figure 2:**
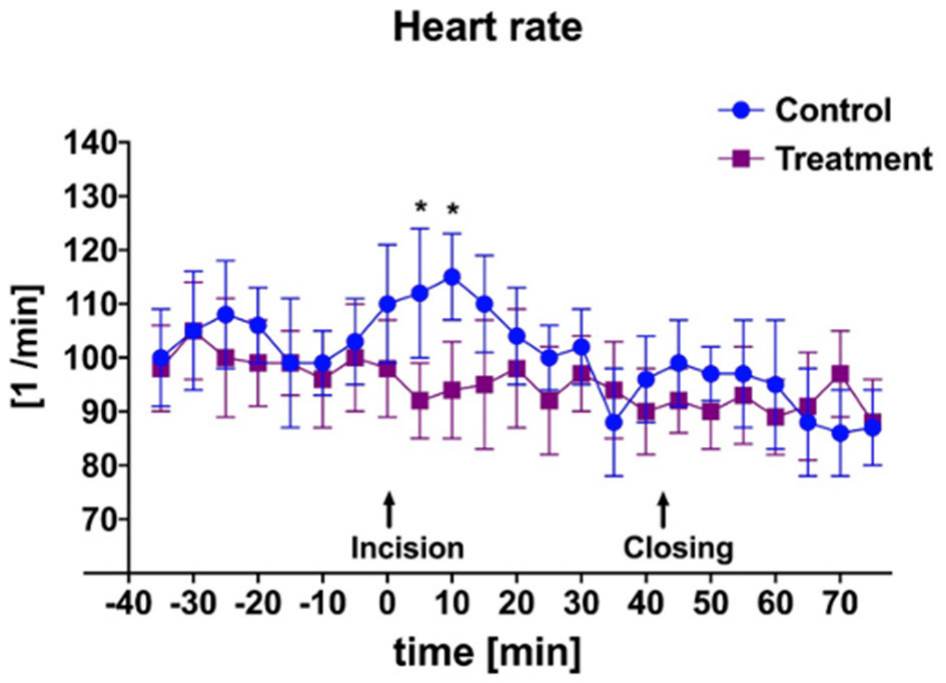
Mean and SD of heart rate throughout anesthetic episode of treatment animals receiving transversus abdominis plane (TAP) block with bupivacaine vs. control animals receiving TAP block with saline. *Data are significantly different *P* < 0.05.

**Figure 3:**
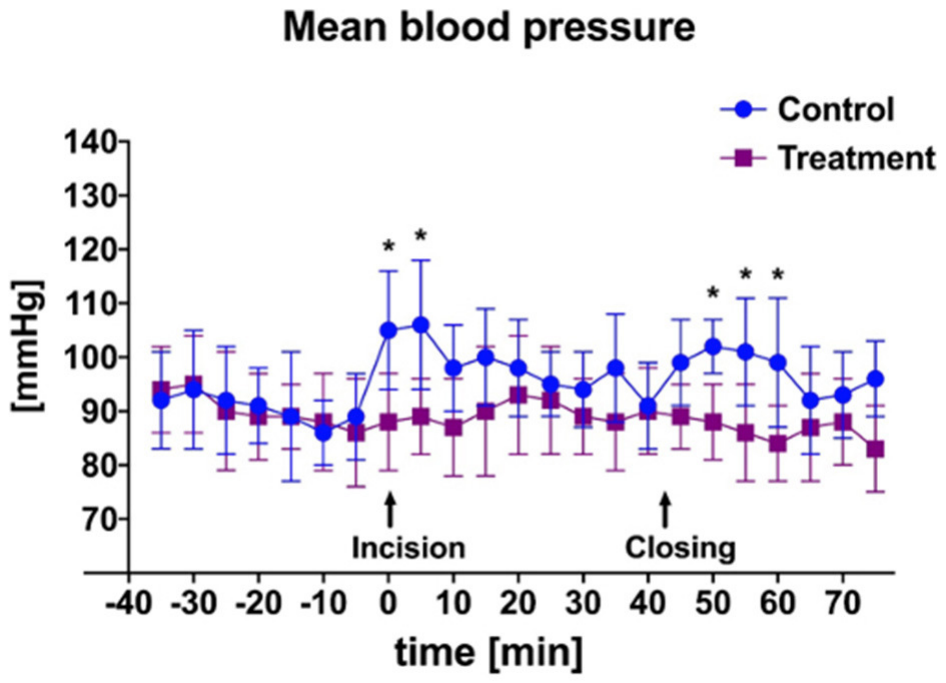
Mean and SD of arterial blood pressure throughout anesthetic episode of treatment animals receiving transversus abdominis plane (TAP) block with bupivacaine vs. control animals receiving TAP block with saline. *Data are significantly different *P* < 0.05.

There was a significant difference in heart rate measured during initial incisional surgical stimulation between groups (Figure 2). At time of start of surgery all goats were mechanically ventilated. Additionally, a significant difference in mean MAP between groups was measured during the periods of incisional and incisional closure surgical stimulation (Figure 3). No goats required cardiovascular support during anesthesia.

**Figure 4:**
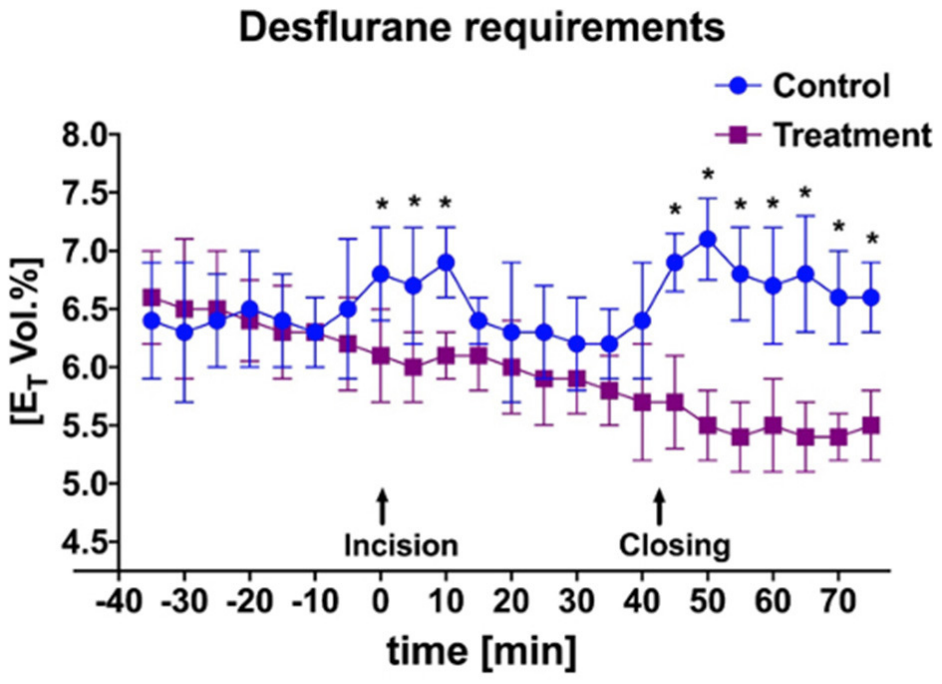
Mean and SD of end-tidal percent desflurane requirement throughout anesthetic episode of treatment animals receiving transversus abdominis plane (TAP) block with bupivacaine vs. control animals receiving TAP block with saline. *Data are significantly different *P* < 0.05.

A significant difference was identified between the E_T_Des requirement during initial incisional and incisional closure surgical stimulation (Figure 4; *P* = 0.03) between the bupivacaine-TAP and control-TAP goats. No significant difference between the numbers of ketamine boluses during surgical stimulation was noted (3 vs. 5 respectively).

No significant difference in total anesthetic time was observed between bupivacaine-TAP (107 ± 11 min) vs. control-TAP (103 ± 14 min) (*P* = 0.68). No significant difference in recovery quality was noticed between groups (*P* = 0.07). A significant difference in recovery time was observed between bupivacaine-TAP (13 ± 8 min) vs. control-TAP (21 ± 15 min) (*P* = 0.03).

**Figure 5:**
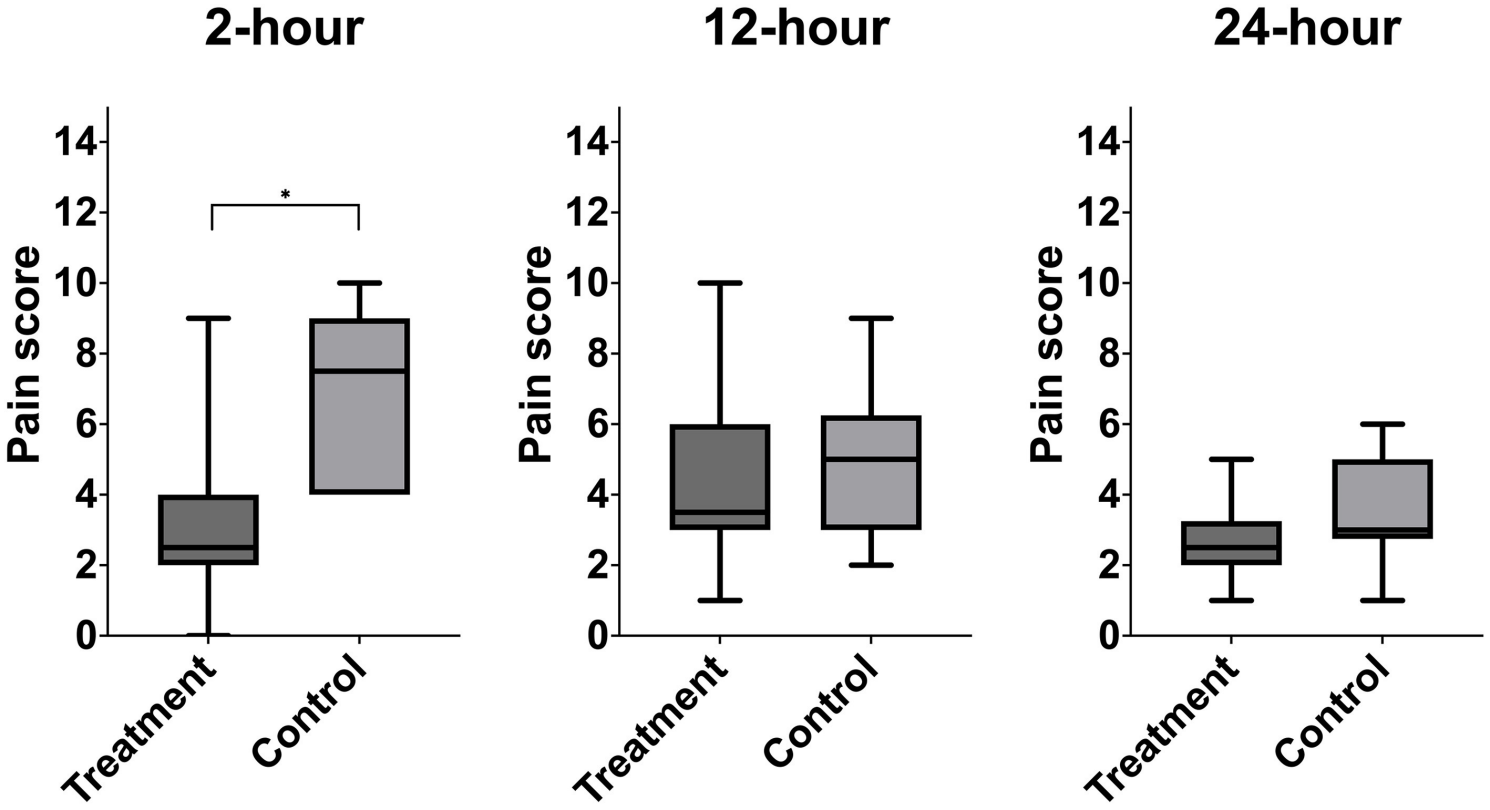
Median postoperative pain score of treatment animals receiving transversus abdominis plane (TAP) block with bupivacaine vs. control animals receiving TAP block with saline. *Data are significantly different *P* < 0.05.

A significant difference in pain score was noted at 2-h post-extubation between bupivacaine- TAP (3, range 0–9) vs. control-TAP groups (7, range 3–10) (Figure 5, *P* = 0.02); however, no significant difference was noted at 12- or 24-h post-extubation (*P* = 0.2 and *P* = 0.09, respectively). A single goat in the bupivacaine-TAP group exhibited a markedly increased 2- and 12-h score (9, 10, respectively) compared to the remaining (median 3, median 4, respectively).

## 4 Discussion

In this study, the use of a TAP block with bupivacaine in goats undergoing ventral midline or paramedian celiotomy was associated with a significant reduction in intraoperative anesthetic requirement during manipulation of the body wall at time of incision and closure. The use of the TAP block was also associated with a significant difference in pain scores between groups at the initial 2-h post-extubation; however, there was no significant difference noted between groups at 12 and 24 h post-extubation. Additional findings included both a reduction in intraoperative mean heart rate and MAP during surgical stimulation (i.e., incision and incisional closure) in addition to reduced recovery time in goats receiving locoregional anesthesia. As such, the benefit of a TAP block in small ruminants undergoing ventral midline or paramedian celiotomy was demonstrated.

Expiratory inhalant anesthetic concentration is an objective means of determining the amount of inhalant anesthetic required to maintain a certain plane of anesthesia and is a surrogate for the alveolar concentration. Thus, it can represent the minimum alveolar concentration, which is the inhalant anesthetic concentration necessary to prevent purposeful movement in 50% of patients in response to a noxious stimulus at sea level (37). Either increasing the alveolar concentration or the total cumulative dose of an inhalant anesthetic predisposes to dose-dependent side effects. The use of local anesthetics as part of balanced anesthetic protocol has repeatedly demonstrated a reduction in inhalant anesthetic required to maintain surgical anesthesia and therefore dose-dependent side effects (38–40). The use of a bupivacaine-TAP block as a part of the anesthetic protocol in goats undergoing ventral midline or caudal paramedian celiotomy supported these earlier findings with a significant decrease in E_T_Des comparatively. One dose-dependent side effect is prolonged anesthetic recovery. Despite no difference in total anesthetic length or recovery quality, bupivacaine-TAP goats recovered significantly faster from general anesthesia suggesting that the recovery length is more so affected by the total anesthetic dose in this population. In combination, the use of local anesthetics to desensitize the surgical site may promote an earlier willingness to stand (41).

An additional finding of this study was the significant difference in both HR and MAP in bupivacaine-TAP vs. control-TAP goats (Figures 2, 3), although these findings were not unexpected. HR and MAP are described markers of nociception in small ruminants that increase in response to noxious stimuli (34, 35, 42). As noted, bupivacaine HCl is a local anesthetic that causes nociceptive blockade. The TAP block effectively desensitizes the ventral abdominal body wall; however, has no analgesic effect on the bladder itself, which is innervated by a combination of the hypogastric, pudendal, and pelvic nerves. No significant rise HR or MAP of the bupivacaine-TAP group was noted during the cystotomy or tube cystostomy portion of the surgical procedure. This phenomenon may be explained by the fact that at least in people, visceral pain is thought more closely associated with distension and that conscious sensation of touch is limited to the urethra and trigone as compared to the apex where surgical manipulation occurs (43). Manipulation of the body wall during this portion of surgery may explain the persistent increase in HR and MAP in the control-TAP group. In following studies it will be useful to compare the effects of spinal analgesia provided to the effects of the described TAP block as spinal analgesia would result in visceral analgesia as well.

In the present study, all goats were evaluated postoperatively using a modified pain scale assigning a numerical value based on assessor's perception of pain, exhibited behaviors and physiological parameters (10, 41, 42). A sum of these values was calculated and used for comparison. The finding of a significant difference in cumulative postoperative pain scores at 2-h post-extubation but no significant difference at either the 12- or 24-h post-extubation time points between groups was not unexpected. The effects of neuraxial bupivacaine in goats undergoing laparotomy and interfascial plane anesthetic blocks in calves and ponies have been reported to last at maximum 12 h and the 12-h post-extubation time point was typically 14 h post-TAP administration (10, 11, 28, 41); therefore, the clinical effect was likely absent at these time points. A feasible alternative to increase the duration of action and therefore prolong the analgesic effects of normal bupivacaine HCl is through the addition of dexmedetomidine to the injectate. Although dexmedetomidine is a highly selective α-2 adrenergic receptor agonist, the prolongation of analgesia is not α-2-mediated. Dexmedetomidine causes dose-dependent peripheral blockade of the inwardly rectifying hyperpolarization-activated cation (I_h_) current responsible for repolarization of the nociceptive type C afferents, thereby prolonging hyperpolarization, and thus nociceptive blockade (44, 45). In humans, dexmedetomidine as an adjuvant to bupivacaine for TAP blocks decreased both the total dose of bupivacaine needed and rescue analgesia requirement as well as prolonged the duration of analgesia (46–48). The use of α-2 adrenergic receptor antagonists at sedative doses in sheep have been associated with severe pulmonary parenchymal damage (49, 50); therefore, they should be used judiciously. Similar effects have not been reported in goats. It is further important to consider that the pain assessment post-operatively included the palpation of the surgery site which could have led to an assessment bias as the incision was likely still desensitized by the TAP block performed prior to starting surgery.

As with all clinical research, limitations exist. On a broader level, first and foremost the TAP block has yet to be described in goats. The combination of a subcostal and lateral TAP block (i.e., two-point) has been described in calves and was extrapolated for this description (26). Although species-specific anatomical variations can exist, the anatomical dissection performed demonstrated similar architecture. This said, a three-point injection technique in cadaver ponies has been shown to be more comprehensive compared to a two-point injection technique regarding nerve staining (28). It is known that ponies have 18 thoracic spinal nerves compared to 13 in both small and large ruminant species; therefore, a two-point technique may be sufficient in these species as the results of this clinical study suggest. There is however potential for a follow-up study comparing differing injection techniques in goats. Similarly, in a retrospective analysis of canines undergoing mastectomy, the TAP block was found insufficient in coverage of the caudal abdomen (16). In goats undergoing celiotomy for obstructive urolithiasis, the incision is cranial to the teat suggesting the coverage of a TAP block should be sufficient in coverage as demonstrated by the results of this clinical study. The authors recognize the potential for lack of coverage in the caudal most ventral abdomen and perineal region; therefore, anecdotally combine the TAP block with an additional locoregional technique (i.e., pudendal nerve block or caudal epidural) when a mastectomy is to be performed in a goat.

Limitations on a granular level with respect to this study, the lack of a uniformed systemic analgesia protocol is one. In the clinical setting, small ruminants with obstructive urolithiasis most often present on an emergent basis and out-of-hours. Therefore, several clinicians with varying experiences and preferences evaluate and treat these animals, which also is a limitation. Furthermore, many of these patients are azotemic at presentation making the use of systemic NSAIDs or corticosteroids with known nephrotoxicity contraindicated. Although, it is argued that the obstructive nature of this disease results in a post-renal azotemia, thus there would be no added risk of perpetuating renal damage with NSAID usage once the obstruction is resolved. This was noted in a single case presenting with azotemia (creatinine 5.2 mg/dL) that was administered a combination of flunixin meglumine and dexamethasone in which following obstruction resolution, the creatine decreased to 1.9 mg/dL within 12 h and 0.8 mg/dL by 36 h. Intraoperative urine specific gravity may be beneficial in determining renal function, assessing dysfunction, and elucidating safety of NSAID use; however, there were no clinically relevant differences in number of animals per group receiving flunixin meglumine and/or morphine analgesia allowing for comparison between groups. The potential variability among those completing the TAP block, administering anesthesia, performing surgery, and making the postoperative pain assessment is another limitation. Using consistent blinded personnel for both the TAP block and pain assessment decreased such inter-person variability, though some mild intra-person variability was likely present. Additionally, the use of a standardized anesthetic protocol with an outlined approach to intervention triggers mitigated this source of variability. Unfortunately, given the nature of a large emergent referral hospital it was not possible to control for multiple treating veterinarians with subtle difference in techniques; however, such were unlikely to affect the outcome or impact of this study. Lastly, there was no objective assessment of accuracy of TAP infiltration with secondary nociceptive blockade. Dependence on the ultrasonographic appearance of hydrodissection within the appropriate fascial plane was used. Case 8 in the bupivacaine-TAP group exhibited a higher pain score compared to all other goats receiving the designated intervention, which could have represented a failure of the TAP block due to the lack of an objective measure of accuracy.

Regardless of these limitations, we found that the use of a transversus abdominis plane block was associated with less intraoperative nociceptive input requiring less inhalant anesthetic leading to faster anesthetic recoveries and decreased postoperative pain scores. As such, it has been adopted into clinical practice at New Bolton Center. Future prospective randomized controlled studies investigating the effect of injectate volume and/or injectate adjuvant (i.e., α-2 adrenergic receptor agonists) to prolong effect are warranted.

## Data availability statement

The original contributions presented in the study are included in the article/Supplementary material, further inquiries can be directed to the corresponding author.

## Ethics statement

The animal study was approved by IACUC University of Pennsylvania. The study was conducted in accordance with the local legislation and institutional requirements.

## Author contributions

KH contributed to study design, data analysis, and manuscript preparation. M-EF contributed to study design and manuscript preparation. TM contributed to study design, data collection, and manuscript preparation. All authors contributed to manuscript revision, read, and approved the submitted version.
