# Supplementary material for: Perioperative analgesic effects of an ultrasound-guided transversus abdominis plane block using bupivacaine in goats undergoing celiotomy
Source: Front Vet Sci. 2023 Nov 24;10:1197728. doi: 10.3389/fvets.2023.1197728 (PMC10704458; doi:10.3389/fvets.2023.1197728)
Supplement: Supplementary file 1 [file Data_Sheet_1.docx]

Supplementary Material

**Perioperative analgesic effects of an ultrasound-guided transversus abdominis plane (TAP) block using bupivacaine in goats undergoing celiotomy**

**Tate B. Morris DVM, DACVS (LA)*, Klaus Hopster DMV, PhD, DECVAA, Marie-Eve Fecteau DVM, DACVIM (LAIM)**

*** Correspondence:**
Dr. Tate B. Morris
tbmorris@vet.upenn.edu

# Supplementary Figures and Tables

## Supplementary Figures


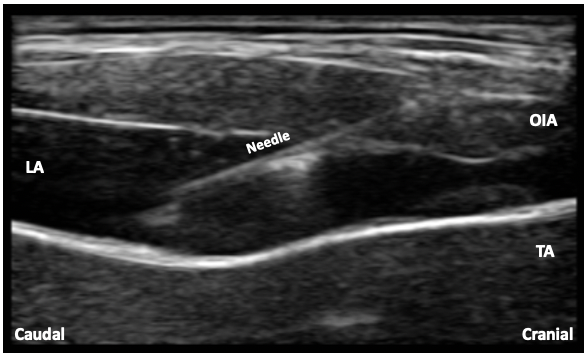


**Supplementary Figure 1.** Magnified sonographic image mid-infiltration of local anesthetic (LA) within the transversus abdominis fascial plane. The linear array ultrasound transducer is oriented cranial-to-caudal parallel to the long axis of the body to a depth of field of 3-5 cm varying based on the individual body wall thickness such that the peritoneal cavity is just visible in the far field. The needle is advanced in a cranial-to-caudal direction 20-30^o^ perpendicular to the skin surface and in plane with the transducer. Note appropriate positioning of needle within the transversus abdominis plane as delineated by the hydrodissection of LA between the more superficial obliquus internus abdominis (OIA) muscle and deeper transversus abdominis (TA) muscle. A volume of 0.4 mL/kg anesthetic solution is infiltrated at each of the four designated sites for injection.

**
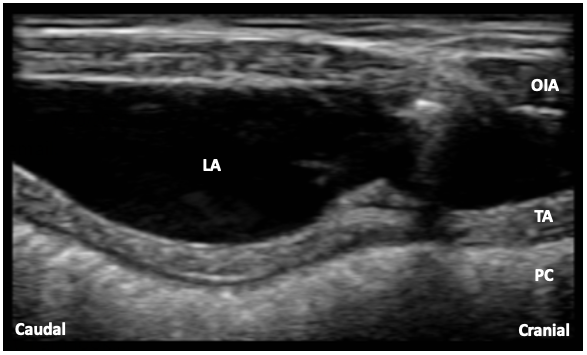
**

**Supplementary Figure 2.** Representative sonographic image post-infiltration of local anesthetic (LA) within the transversus abdominis fascial plane. The ultrasound transducer is oriented cranial-to-caudal parallel to the long axis of the body. Note the hydrodissection between the more superficial obliquus internus abdominis (OIA) muscle and the deeper transversus abdominis (TA) muscle. The peritoneal cavity (PC) with gastrointestinal viscera is seen in the far field.


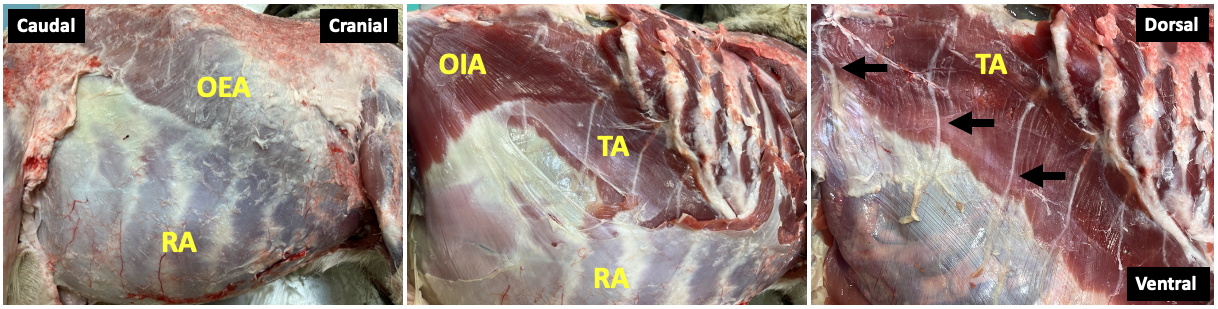


**Supplementary Figure 3.** Sequential superficial-to-deep anatomical dissection images depicting the dorsal overlying obliquus externus abdominis (OEA) and ventral rectus abdominis (RA) muscles (left image). Reflection of the OEA exposes the underlying obliquus internus abdominis (OIA) muscle as well as the underlying transversus abdominis (TA) muscle depending on individual anatomic variation of the OIA muscle belly development (middle image). Reflection of the OIA and RA muscles exposes the ventral branches of the thoracolumbar spinal nerves (black arrows) coursing perpendicular to the long axis of the body superficial to the TA muscle within the transversus abdominis fascial plane (right image).


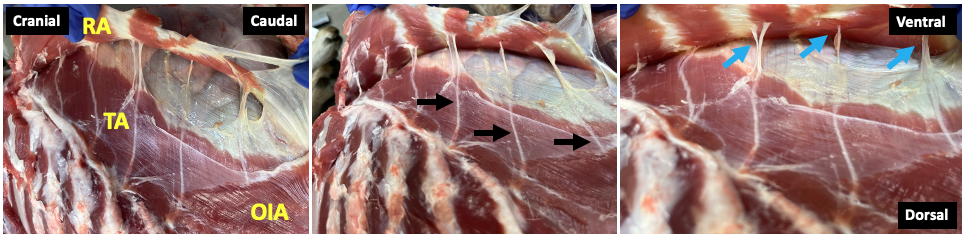


**Supplementary Figure 4.** Sequential magnified anatomical dissection images depicting the ventral branches of the thoracolumbar spinal nerves (black arrows) as they course within the transversus abdominis fascial plane perpendicular to the long axis of the body deep the obliquus internus abdominis (OIA)/rectus abdominis (RA) muscles and superficial to the transversus abdominis (TA) muscle. Note the termination of the nerves within the deep margin of the RA muscle (blue arrows).

**
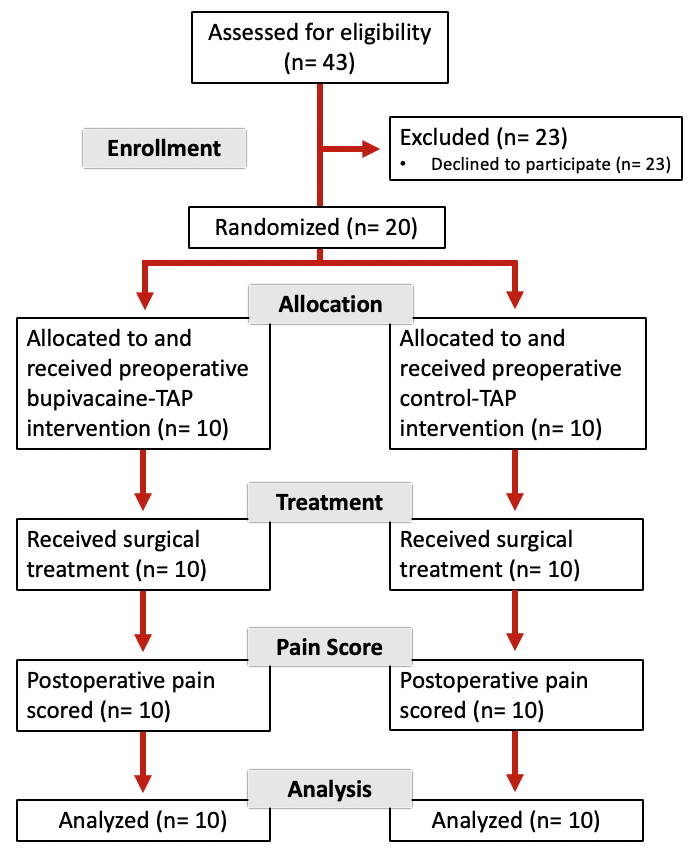
**

**Supplementary Figure 5.** CONSORT flow diagram of goats presenting for surgical treatment of obstructive urolithiasis at the Widener Hospital of the School of Veterinary Medicine, University of Pennsylvania between October 2019 and August 2020. Study enrollment was voluntary based on owner preference, clinician participation, and available resources on an emergency basis.
